# Supplementary figures and images for: What constrains food webs? A maximum entropy framework for predicting their structure with minimal biases
Source: PLoS Comput Biol. 2023 Sep 5;19(9):e1011458. doi: 10.1371/journal.pcbi.1011458 (PMC10503755; doi:10.1371/journal.pcbi.1011458)

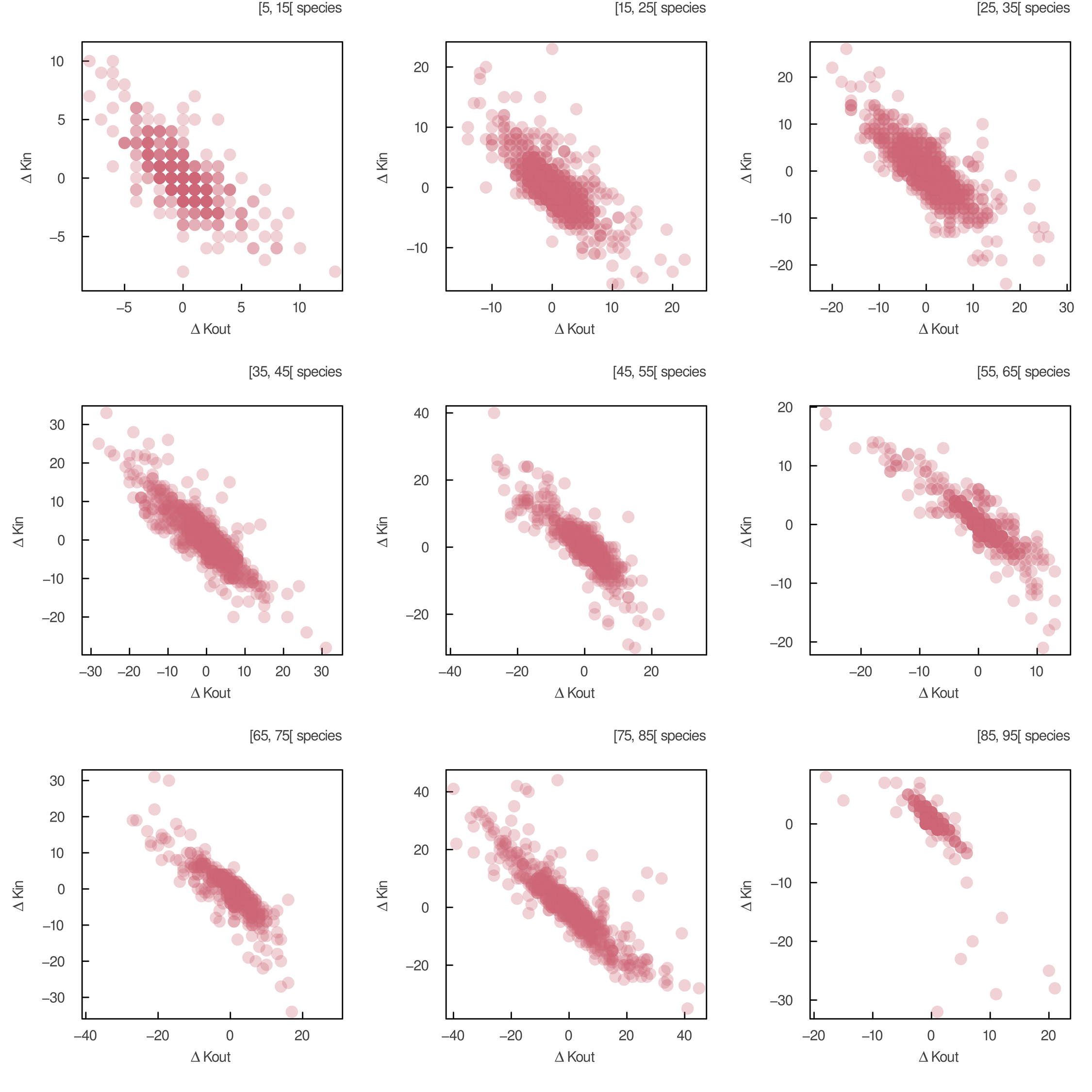

Supplement: S1 Fig — Species were ordered according to their total degree in their network. Networks were sorted into different groups based on their total number of species. In each panel, each dot corresponds to a single species within one of the networks whose total species count is within the specified range. The predicted joint degree sequences were obtained after sampling one realization of the joint degree distribution of maximum entropy for each network while keeping the total number of interactions constant. (TIF) [file pcbi.1011458.s001.tif]

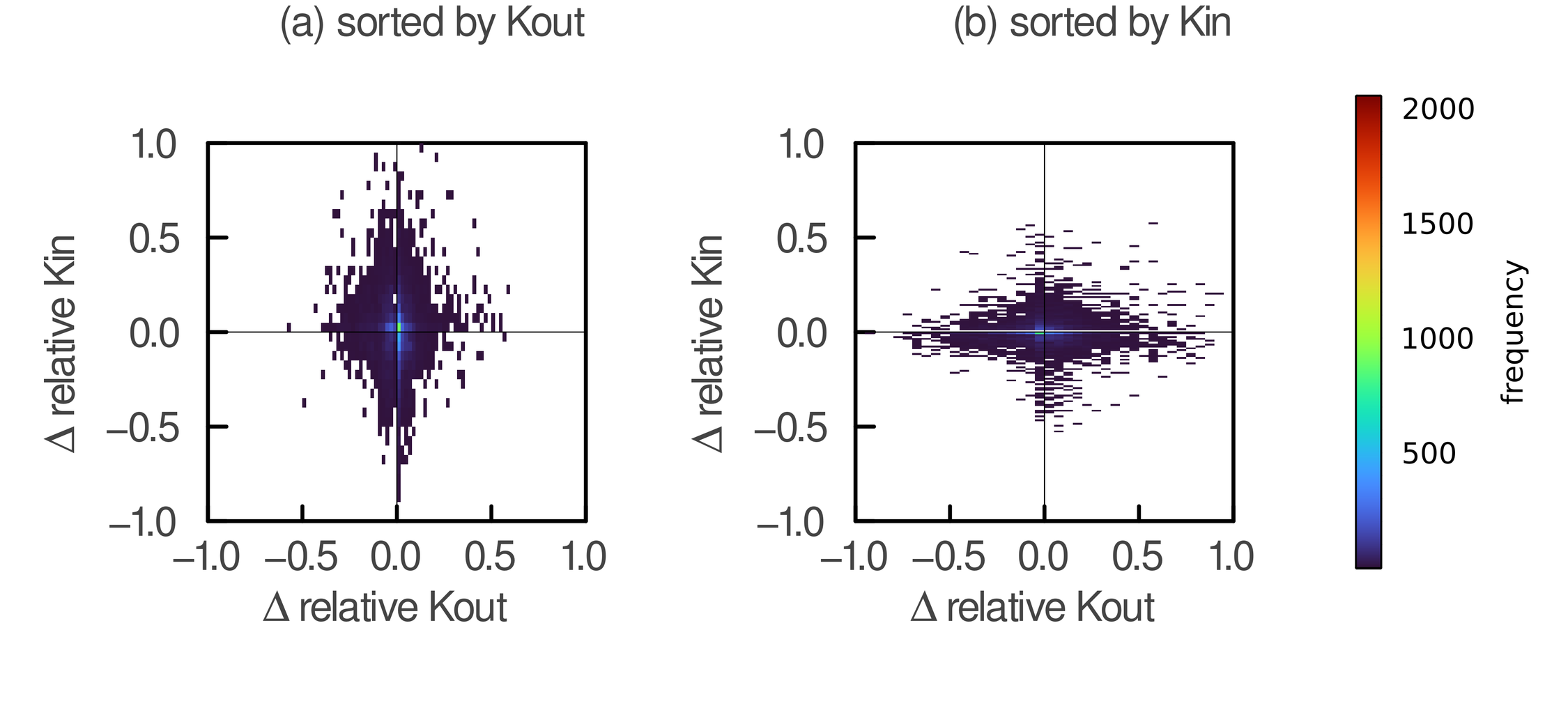

Supplement: S2 Fig — Species were ordered according to (a) their out-degree and (b) their in-degree. The predicted joint degree sequences were obtained after sampling one realization of the joint degree distribution of maximum entropy for each network while keeping the total number of interactions constant. Due to significant data overlap, all relationships are represented as 2D histograms. The color bar indicates the number of species that fall within each bin. (TIF) [file pcbi.1011458.s002.tif]

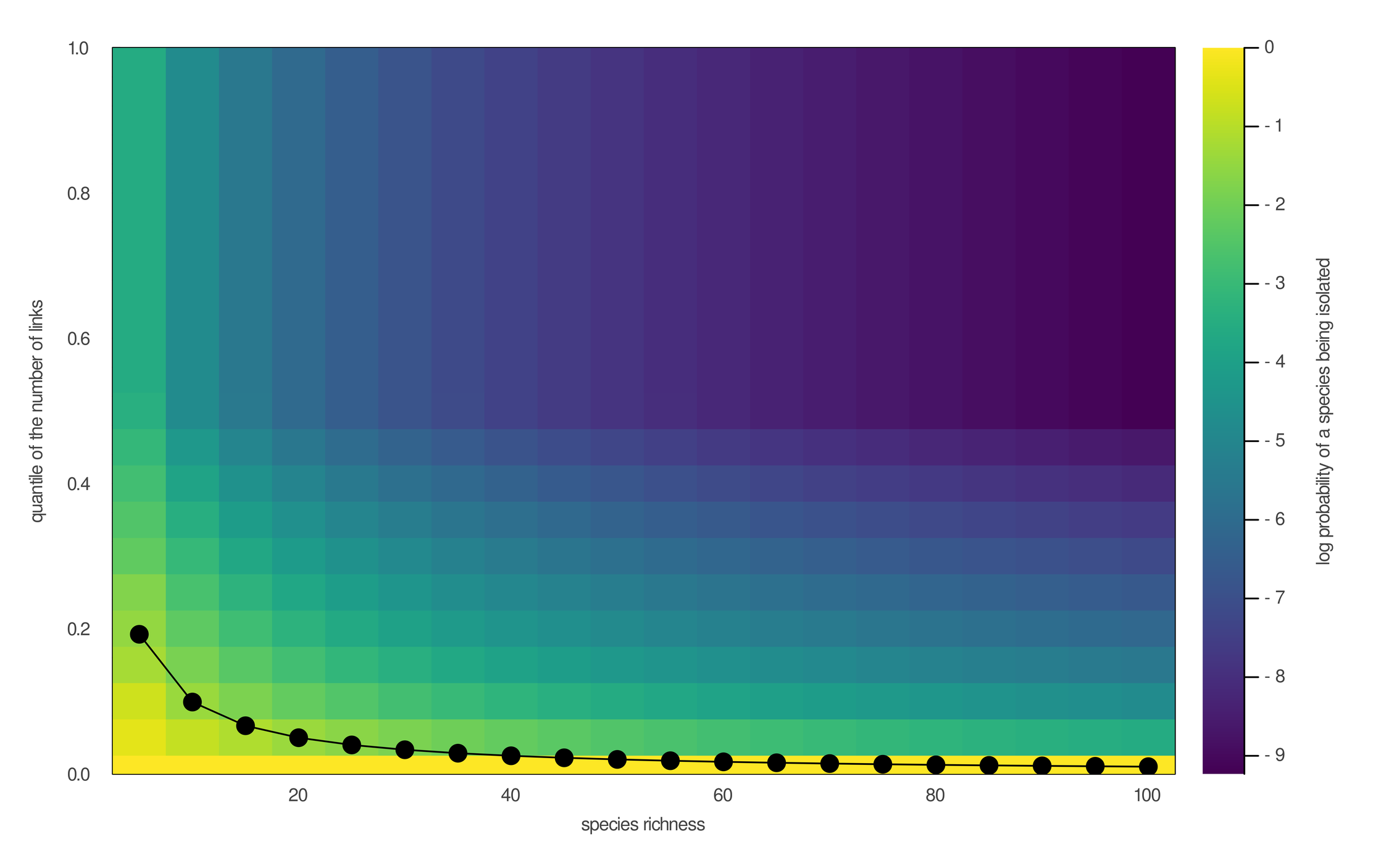

Supplement: S3 Fig — We derived many degree distributions of maximum entropy given a range of values of S and L and plotted the probability that a species has a degree k of 0 (log-scale color bar). Species richness varies between 5 and 100 species, by increment of 5 species. For each level of species richness, the numbers of interactions correspond to all 20-quantiles of the interval between 0 and S2. The black line marks the S − 1 minimum number of interactions required to have no isolated species. (TIF) [file pcbi.1011458.s003.tif]

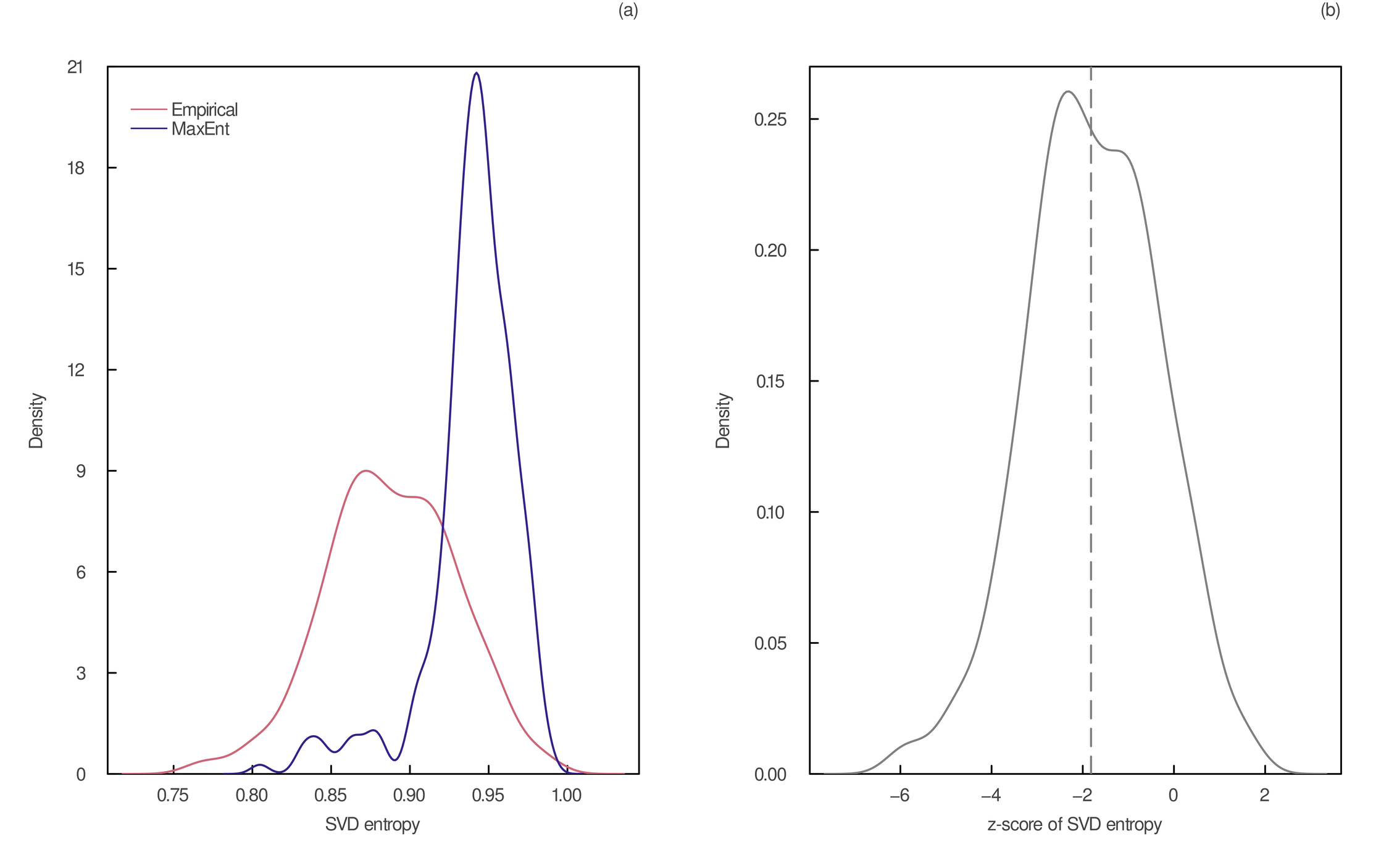

Supplement: S4 Fig — (a) Distribution of the SVD entropy of empirical and maximum entropy food webs. Maximum entropy networks were obtained using the type II heuristic MaxEnt model based on the joint degree sequence. (b) Distribution of z-scores of the SVD entropy of all empirical food webs. Z-scores were computed using the mean and standard deviation of the distribution of SVD entropy of MaxEnt food webs (type II heuristic MaxEnt model). The dashed line corresponds to the median z-score. (TIF) [file pcbi.1011458.s004.tif]

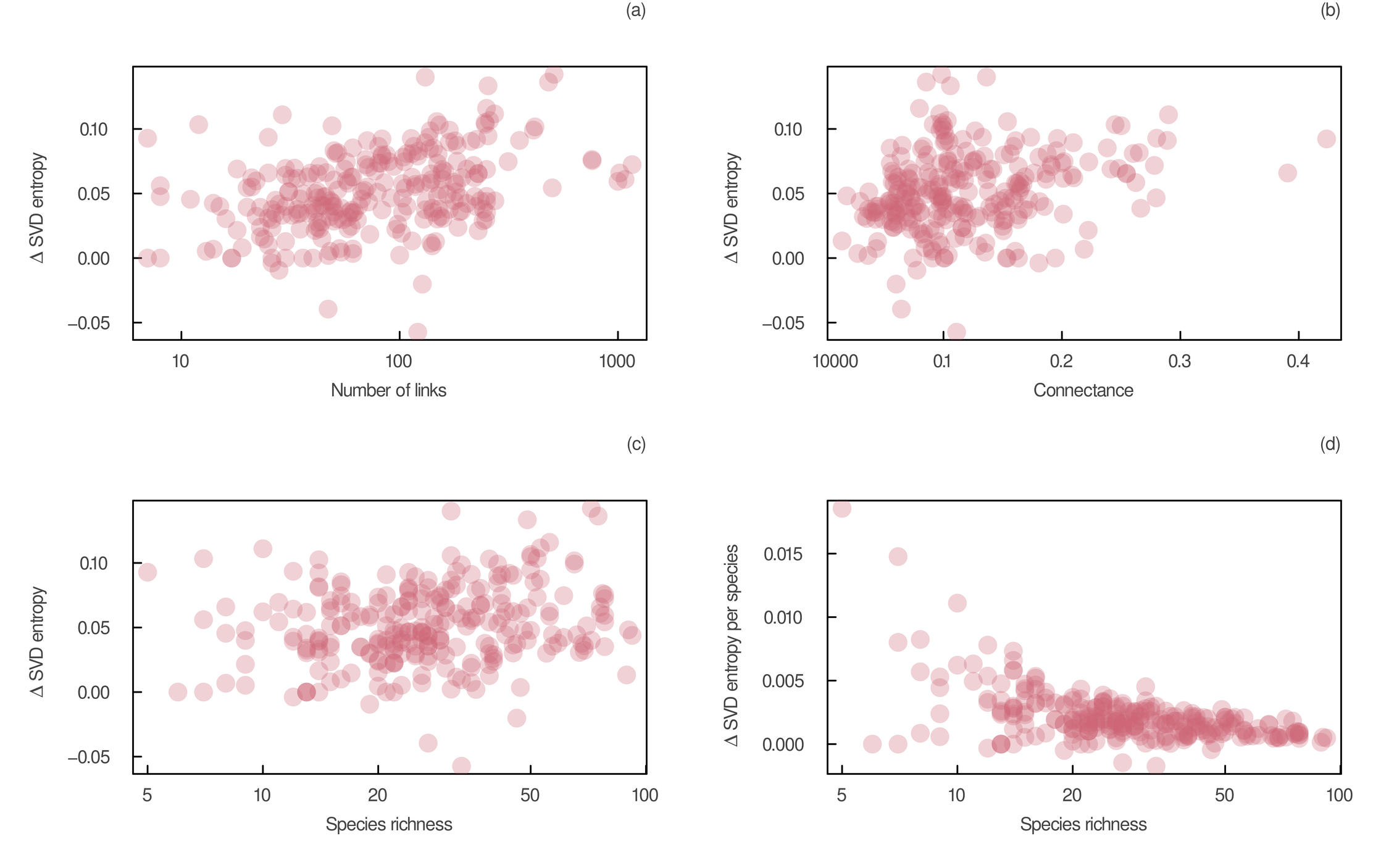

Supplement: S5 Fig — Difference in SVD entropy between maximum entropy and empirical food webs as a function of (a) the number of interactions, (b) connectance, and (c) species richness. (d) Standardization of the difference in SVD entropy with respect to species richness as a function of species richness. The exponential decrease in the difference of SVD entropy per species with species richness offers a complementary perspective supporting the lack of relationship depicted in panel c. Maximum entropy networks were obtained using the type II heuristic MaxEnt model based on the joint degree sequence. (TIF) [file pcbi.1011458.s005.tif]

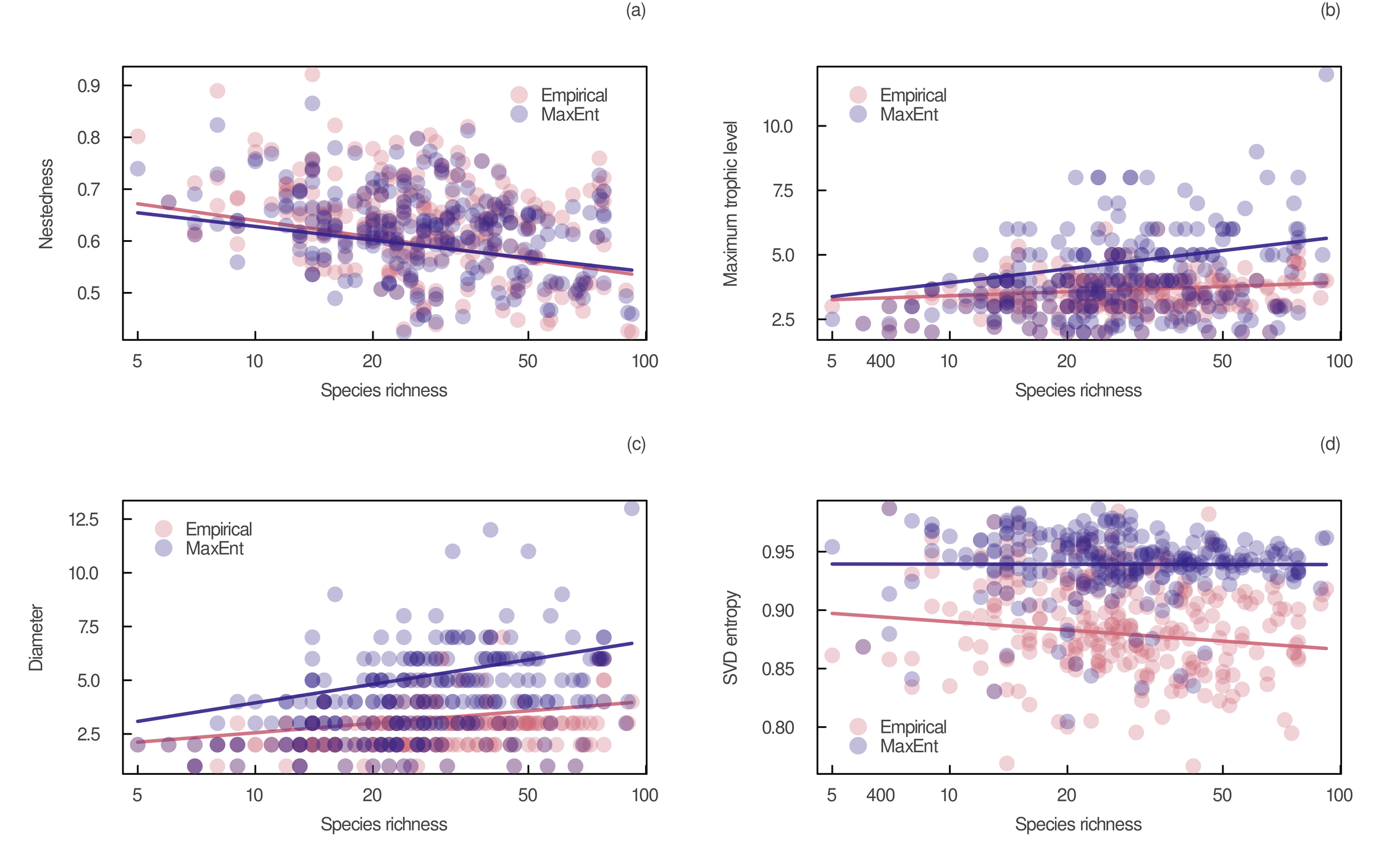

Supplement: S6 Fig — Maximum entropy networks were obtained using the type II heuristic MaxEnt model based on the joint degree sequence. (a) Nestedness (estimated using the spectral radius of the adjacency matrix), (b) the maximum trophic level, (c) the network diameter, and (d) the SVD entropy were measured on these empirical and maximum entropy food webs and plotted against species richness. Regression lines are plotted in each panel. (TIF) [file pcbi.1011458.s006.tif]
