# Supplementary material for: What constrains food webs? A maximum entropy framework for predicting their structure with minimal biases
Source: PLoS Comput Biol. 2023 Sep 5;19(9):e1011458. doi: 10.1371/journal.pcbi.1011458 (PMC10503755; doi:10.1371/journal.pcbi.1011458)
Supplement: Attachment — Submitted filename: response_PLOS.pdf [file pcbi.1011458.s007.pdf]

Chaitanya S. Gokhale, PhD
Natalia Komarova, PhD
Academic and Section Editors
PLoS Computational Biology
1265 Battery Street, Suite 200, San Francisco, CA 94111, USA

July 13th, 2023

Dear Drs. Gokhale and Komarova,

We would like to thank you for your valuable feedback on our manuscript "What constrains food webs? A maximum entropy framework for predicting their structure with minimal biases". We are pleased that you and the reviewers found interest and worth in our work. We are confident that the manuscript has been significantly improved after addressing each suggestion and hope that it will now be approved after your consideration. We have clarified many technical terms, provided more explanations about our methods and reasoning, and fixed grammatical errors. Additionally, we have improved our first figure (now our second figure) and reran simulations as suggested by the reviewers. Finally, we have modified the organization of the manuscript to comply with PLoS guidelines, i.e. by creating and moving content to the Methods and Results & Discussion sections. We have included the Methods before the Results to bring this important section to the forefront and maintain a smooth flow of ideas when presenting our framework. As requested, we have uploaded a clean version of the revised manuscript and a version highlighting the modifications made in the text. Our changes and responses to the reviewers' comments are detailed below, with new line numbers referring to the revised PDF file.

Reviewer #1:

The manuscript "What constrains food webs? A maximum entropy framework for predicting their structure with minimal biases" presents an elegant strategy to develop null models to describe food networks. The advantage of the proposed strategy, relying on the maximum entropy framework, is its simplicity and versatility, nicely boosted by the possibility of using only S, the number of species, to generate null predictions about

several features of the networks. For food networks, null models already exist, but the ones presented in this manuscript seem to capture better the properties of empirical food networks; in addition, I'd argue that the maximum entropy framework presents a more solid foundation for such null models. Finally, the paper describes the main framework very clearly, and it is well written. Thus, I am sure it will allow ecologists not versed in information theory to implement such null models easily. Therefore, I welcome this paper and I recommend its publication in the present form, with only minor adjustments. I only have a few minor comments, mostly related to the form and the comparisons with the empirical data, which I think could be improved.

We thank the reviewer for their encouraging comments. We have improved the graphical representation and layout of Fig 1 (now Fig 2) and provided more explanations and greater context to our results, as suggested. We are confident that our adjustments have rendered the comparisons with empirical data clearer and that they facilitate their interpretation.

Motif profiles are not described at all in the text, nor in the figure caption. The authors refer to Stouffer 2007, but I think that adding a minimal description would significantly improve readability. This is important because I think some of the strongest points in using MaxEnt2 and using joint degree sequence as constraint is made by figure 5 and 6. It is nice how MaxEnt2 seems to predict better and more consistently all motif profiles. However, at the moment, these are not completely transparent.

We have provided a more thorough definition of motifs in the first paragraph of the section on the structure of MaxEnt food webs and described motif profiles subsequently (lines 375-380). We have also described each of the single-link motifs S1-S5 in the captions of Figs 5 and 6 and slightly reformulated the paragraph discussing these results (lines 604-624). We agree with the reviewer that adding a more detailed explanation of motifs was necessary to improve readability and help readers understand better the implications and significance of our results.

For instance, in figure 6 I notice that motif S3 is not shown. Is that because of the low total abundance?

Yes, we decided not to show motif S3 in Figure 6 because of its very low proportions in most empirical and predicted networks. For instance, we found it in only 4 empirical food webs. We have added a short sentence in the figure caption to explain this.

> *Figure 1 could be improved. There is a lot of overlap of points, maybe a heatmap or a different representation could be better. Also the layout, spacing and so on could be improved. I also understand why normalizing relative Kout and Kin, but perhaps there is enough space (if not, in the supplementary) to stratify by connectance or S, particularly figure c.*

As suggested by the reviewer, we have represented all panels of Fig 1 (now Fig 2) and S2 as 2D histograms to represent more clearly the number of points that fall within each bin and changed the layout of the figure to optimize the spacing between panels. Moreover, we have added a figure in the supplementary material (Fig S1) illustrating the relationship depicted in Fig 2C for absolute (non-relative) values of Kout and Kin, stratifying networks by varying levels of species richness (described on lines 490-492). This novel figure provides a similar result as in Fig 2C, but offers a lens through which to interpret it in light of species richness. We decided to represent Fig S1 as a series of scatter plots given that the stratification reduced the overlap of points.

> *The sentence "Empirical networks include most food webs archived on Mangal, as well as the New Zealand and Tuesday Lake datasets (our complete dataset)" is repeated over and over in every caption. I don't think it is needed since all analyses are done on that dataset. Expect table 2?*

This sentence was repeated to avert any potential confusion between the two datasets. After reconsideration, we agree that it hampered the readability of the text and might not be needed to understand the figures correctly. We have removed this sentence from the figure and table captions. However, we kept the datasets' names in both table captions to highlight their differences and enhance clarity.

Weird phrasing in line 416 ("We found no to a weak..").

We have reformulated this sentence (lines 585-587).

I think it would also be nice if the authors could comment more on Fig.S4, which seems to show the fact that SVD Entropy is more poorly predicted for larger networks. Perhaps one should standardize this somehow?

Our interpretation of the results depicted in Fig S4 (now Fig S5) is different from the one of the reviewer. We have clarified our understanding of our results by rewording the phrasing in line 416 (now lines 585-587) and removing the regression lines that misleadingly suggested a relationship between the predictive errors of SVD and network size and order. We have expanded the discussion of Fig S5 by elaborating on the fact that our model predicted complexity similarly well for both small and large networks (lines 585-592). Finally, based on the reviewer's suggestion, we have standardized SVD entropy by adding a panel in Fig S5 showing the relationship between the difference in SVD entropy *per species* and species richness. This panel provides an alternative way of displaying the lack of relationship between the prediction of complexity and network size.

I also think it is quite nice that the authors show that their null models predict very well several features of food webs, motif profiles, nestedness, etc. Given this result, it is quite interesting that instead entropy SVD-entropy itself is less well predictable, especially since it is a at the core of the model. I wonder if this means that motif profiles as currently defined are not that biologically relevant, and are merely a statistical property of these networks. Could the authors reflect on this, and maybe add a line or two in the discussion about potential applications.

The low predictability of entropy may be an indication of additional constraints shaping food-web structure. Indeed, the maximum entropy value gets closer to the entropy of empirical networks as more constraints are added to the model. This is what we show when we present the improved capacity of the MaxEnt model based on the joint degree sequence to predict entropy

compared to the one solely based on connectance. We have developed our interpretation of this result in the paragraph discussing the prediction of entropy (lines 572-585) and added a sentence in the section on potential applications of MaxEnt as a null model, as suggested (lines 727-729). Moreover, we have enriched the discussion of our results on the three-species motifs by suggesting that they may be a statistical attribute of networks because of the ability of MaxEnt to adequately reproduce motif profiles from the joint degree sequence (lines 621-624).

Reviewer #2:

The article delves into a crucial topic in ecology, which is essential to comprehend and predicting ecological networks. It thoroughly discusses MaxEnt, a model that proves to be a valuable tool for ecologists, allowing researchers to understand connectance and ecosystem structure better. The authors explain the applications, motivations, and mathematical formulas clearly and concisely. Their findings are significant and present an exciting approach to their use in real-world data, hence having great applicability in conservation. However, the article could benefit from more elaboration on technical terms to help non-specialised readers.

We express our gratitude to the reviewer for their motivating and supportive remarks. We have greatly elaborated on technical terms to make our manuscript more accessible to a broader readership.

9-12. The knowledge gaps surrounding web properties need to be addressed. The author's reasoning for the complexity of ecological network structure and behaviour needs to be clarified. Consider rephrasing the sentence.

We have discussed the knowledge gaps surrounding network structure and rephrased these sentences to clarify our reasoning (lines 13-21).

16-17. Define nodes and edges in an earlier moment.

We have defined nodes and edges at the beginning of the first paragraph, along with connectance (lines 6-9).

17. Remove "indeed."

Removed (line 30).

17-22. The sentences seem disconnected. Rephrase.

We have rephrased a significant portion of this paragraph to facilitate the smooth transition of ideas (lines 28-35).

24: Define "maximum trophic level" earlier in the text.

We have defined the concept of “maximum trophic level” after mentioning it for the first time in the second paragraph (lines 36-37).

25. Remove "thus".

Removed (line 39).

26. Remove "indeed."

Removed (line 40).

26. Define "Eltonian shortfall."

We have defined the concept of Eltonian shortfall (lines 41-42).

28. Please expand on how network constraints could be used to predict pairwise interactions and network properties. Especially they might be relevant to the maximum entropy framework.

We have reshaped this paragraph and significantly expanded our discussion on how network constraints can alleviate the Eltonian shortfall (lines 43-58).

34. Remove "thus".

Removed (line 60).

34-35. The text needs to clarify the distinction between understanding and predicting.

We have significantly restructured this paragraph to clarify the distinction between understanding and predicting. In particular, we have provided a more thorough definition of predictive and null models and compared their use in network ecology more precisely (lines 60-80).

36. Define "null models" before discussing their benefits.

We have defined null models more thoroughly before discussing their benefits (lines 69-78)

37-38. Needs clarification.

We have provided more details on the functioning of null models (lines 69-76).

40. Define "predictive models".

We have defined predictive models more thoroughly (lines 60-64).

52-52. Please expand on this.

We have expanded our short example by clearly defining the species abundance distribution and identifying the state variables and constraints (lines 92-99).

> *56-57. Is there a particular reason on why entropy would have a different definition from physics (i.e. uncertainty of a random process)? If so, the reader might benefit from knowing, especially when a widely known term is redefined.*

We have redefined entropy as suggested by the reviewer to reduce any ambiguity (lines 103-104).

> *61. Is "discrete case" an essential aspect of this sentence? Discrete can be a data property, and it might confuse readers. Rephase to " a measure of entropy should satisfy four properties:"...*

We have rephrased this sentence as suggested by the reviewer (line 107). We originally specified that it was in the discrete case because we defined Shannon's entropy for a discrete random variable (i.e. for the numbers of predators and prey). However, the four properties of entropy could also be applied to a continuous random variable. We removed this specification to prevent any confusion among the readers.

> *84. Nodes and edges should have been defined earlier in the text.*

We have defined nodes and edges at the beginning of the first paragraph, along with connectance (lines 6-9).

> *103-104. Clarify the choice to use deterministic and unweighted food webs.*

We have clarified the reasons why we used deterministic and unweighted food webs (lines 164-170).

> *107-110. Needs clarification.*

We have reformulated the explanation of the calculation of the degree distribution from the joint degree distribution (lines 176-178).

> *114. Needs citation.*

We have cited the paper that proposed the current name of the simulated annealing algorithm and compared it with annealing in solids (line 184).

> *137-148. In complex food webs, such as the data employed, joint probability distribution is often miscalculated given the number of interactive components in the system. How does this noise affect MaxEnt estimates?*

The reviewer raises a good point about the miscalculation of network properties such as the joint degree distribution in empirical data. Our MaxEnt models aim at reproducing the sampled structure of networks, not their actual structure (i.e. if interactions were comprehensive). In other words, they use the values of the state variables measured empirically to infer distributions of sampled interactions. We have clarified this point right after describing the data (lines 225-229). MaxEnt could be used to infer actual joint degree distributions by using more precise estimates of the state variables, but this is beyond the scope of this manuscript.

> *198-199. Can you expand on joint degree measurements versus individual measurements? Also, if an element (predator or prey) is present in multiple sequences, would this be accounted for repeatedly? If so, would this affect the prediction estimates of a network?*

We have slightly rephrased this sentence to ensure clarity between individual and joint degree measurements (lines 479-484). Because we predicted a distribution of pairs of values (a joint distribution), species are not accounted for repeatedly. Put differently, if there are S species in a network, we will have S different predicted pairs of values (a number of prey and predators for each species). However, we do not directly know which species each pair of values is predicted

for (since we predicted the entire joint degree sequence without taking into account species identity, solely based on S and L). We had to associate each predicted pair of values to a species to make species-level comparisons.

> *213-216. Needs clarification.*

We have rephrased our explanation of our methods and provided more details on the measure of KL divergence (lines 504-509). We have also cited the paper describing this measure (line 505).

> *230. Remove "indeed"*

Removed (line 281).

> *263. Why a beta-binomial distribution? Stable interaction webs usually have an exponential distribution. Clarify if the distribution choice is due to the results in figure 3.*

We have clarified our choice of using a beta-binomial distribution to predict the number of interactions in food webs (lines 400-412). Our choice was informed by our knowledge of the ecological processes driving the total number of links in a food web and by the high predictive capacity of the flexible links model of MacDonald *et al.* (2020), which is based on the beta-binomial distribution. The results in Fig 3 (now Fig 1) are obtained after generating data on the number of links using this model but did not impact our choice of distribution.

> *267. Monte Carlo simulation describes an arbitrary interaction process. Would the algorithm choice influence the determination of a non-arbitrary process, such as food networks?*

Monte Carlo simulations were used to sample model parameters from the posterior distribution. The non-arbitrary ecological processes driving the number of interactions in food webs are represented by the model (i.e. the choice of distribution, number of trials, and parameters), but

the algorithm used to infer the posterior distribution has to be stochastic to ensure proper sampling.

268. Why a single chain? Simulations usually have 2+ to ensure proper mixing.

We have rerun our simulations using 4 chains (line 417). Even though the predicted numbers of links shown in Fig 1 slightly changed, our results remained qualitatively the same.

310. Remove "indeed"

Removed (line 297).

316. Define the acronym "SVD" in an earlier moment.

We have defined the acronym SVD at the end of the first paragraph of this section (line 303), just before describing it in the following subsection on SVD entropy.

317-329. That is a great definition of the MaxEnt process. The authors might consider moving this session to when MaxEnt was first introduced.

We appreciate the reviewer's positive comment. We considered moving this section to the suggested location, but we finally decided to leave it untouched in its current place. We believe that it serves as a good introduction to our second approach and that it could confuse readers if presented earlier in the text. However, we have added two sentences in the section where MaxEnt was initially introduced to include the possibility of predicting the adjacency matrix to the other examples of food-web properties that can be derived using MaxEnt (lines 144-148).

366. Define "motifs"

We have provided a more thorough definition of motifs and motif profiles in this paragraph (lines 375-380). We have also described each of the single-link motifs S1-S5 in the captions of Figures 5 and 6.

333. Define SVD.

We have provided additional clarifications on how SVD operates (lines 327-329).

421. Remove "indeed"

Removed (line 595).

Reviewer #3:

Briefly, it is my opinion that this paper should be published with minor revisions. It adds to a growing body of literature on MaxEnt work within ecology and makes important contributions towards predicting food web structure.

We want to thank the reviewer for their kind words throughout this review.

The authors leverage the principal of maximum entropy to find the least-biased probability distribution given the constraints of a food web. This least-biased probability distribution is the one with the highest entropy among all probability distributions that satisfy the given constraints. The authors chose to use two complementary approaches to predict the structures of food webs using the maximum entropy principle. The first approach consists of deriving constrained probability distributions of given network properties analytically, while the second approach consists in finding the adjacency matrix of maximum entropy heuristically. These attempts at predicting food web structures through the principle of maximum entropy are novel and build the groundwork for the use of MaxEnt in food web studies.

The reviewer provided a concise and accurate summary of our study.

> *The authors found mixed results when attempting to predict food web structures with MaxEnt. They found that MaxEnt predicts the joint degree distributions well in high-connectance systems, but when connectance was low MaxEnt distributions were too symmetrical. In their heuristic approach, they found that MaxEnt models based on connectance did not predict food web structure very well. They did, however, find that the heuristic model based on joint degree distributions reproduced food web structure quite well. Nevertheless, this approach was still not able to predict realistic food-chain lengths.*

This is exact. We wanted to show the iterative process of building MaxEnt models that can reproduce network structure. Identifying which network properties are poorly predicted by a MaxEnt model can guide the development of other models by hinting at additional constraints that could be incorporated. This is what we did when we constrained the adjacency matrix with the joint degree distribution after analyzing the predictions of the simpler heuristic model based on connectance. These deviations are also ecologically meaningful on their own because they inform us about potential mechanisms that can drive food-web structure beyond the constraints used.

> *The authors do a good job grounding this study in MaxEnt, network, and food web literature. They complete multiple tests on both of their approaches, running their MaxEnt models against null models, neutral models, and against empirical food webs. In novel research like this, running many tests is important to show where this strategy works and where it does not. While their results show that MaxEnt may not be ideal for certain predictions of food webs with specific constraints such as low-connectance, they did find that in high-connectance systems the analytical method works well for predicting joint degree distributions. This paper builds the basis for the application of the principle of maximum entropy for predicting food webs. I believe this is an important contribution to the future of food web studies, both the successes and failures of their MaxEnt predictions.*

We appreciate the reviewer's kind remarks. Conducting numerous tests is key to gaining a proficient understanding of the capacity of MaxEnt models to reproduce network structure. The successes and failures of our MaxEnt models can provide insights for subsequent applications of the principle of maximum entropy for predicting food-web structure.

> *Overall, this paper is well written and shows how novel research may not produce expected outcomes, but that certain aspects can be used for future applications. This paper makes an impact in the way that it lays the foundation to use MaxEnt principles for structural predictions of food webs. I recommend publication with (very) minor revisions.*

Again, we are grateful to the reviewer for their uplifting words. We believe that showing how to assess and interpret the limits of MaxEnt predictions is an important part of a manuscript that aims at supporting the use of this method to predict and test the drivers of food-web structure.

> *I suggest that on line 263 when discussing the beta-binomial distribution that the authors make note that "BB" in the following two equations (16 & 17) represent the beta-binomial distribution. That could be represented in the sentence as such, "...obtained from a beta-binomial distribution (BB)..." just to clarify for the readers who may not be familiar with beta-binomial distributions. I recognize that "BB" is a standardized scientific nomenclature, however, it would be helpful to explain it in its first usage. The details of the methodology are, as mentioned, easy enough to follow which allows for this project to be replicated.*

We have introduced the notation of the beta-binomial distribution as suggested by the reviewer (line 411). We agree that this precision contributes to a better understanding of both equations.

> *I might also suggest a glance at Caruso et al. (2022) "Fluctuating ecological networks: A synthesis of maximum-entropy approaches for pattern detection and process inference" as a paper to cite when referencing MaxEnt approaches in ecology. This is another paper that analyzes the ability to use MaxEnt with the constraints of ecological networks. This paper does not look specifically at food webs; however, it does discuss the applications of*

MaxEnt in ecology. So, it might be pertinent to include this new research in your citation list.

We would like to thank the reviewer for bringing this fascinating reference to our attention. We have cited this paper in the introduction when presenting the use of MaxEnt in ecology (lines 124-126) and in the conclusion when discussing how our method can be used to generate null models (lines 724-727).

Line 254: "However, since the later is rarely measured empirically…" I believe this should be "latter" instead of "later".

We have fixed this grammatical error (line 396).

Line 309: "This type of models…" I believe models should either be singular or "this type" should be plural.

We have fixed this grammatical error by singularizing the expression (line 296).

The authors meticulously broke down their methodology and the equations associated with their predictions. I personally found it easy to follow their succession of equations, which build on one another throughout their methodology sections.

We are pleased that the reviewer found the equations easy to follow. We wanted to present the approach thoroughly using a succession of equations so that readers could derive their own using other constraints on the structure of food webs or other biological networks.

The authors' inclusion of potential future applications of this work supports their findings and demonstrate this paper's function as footing for using MaxEnt in food web studies. They noted where it works when predicting food webs and where it does not. This is important information when discussing potential future applications for MaxEnt in food webs research. The authors lay out four specific potential applications for MaxEnt in

food web structural research. This paper tackles the important task of deciphering state variables that do and do not reproduce empirical data.

We agree that discussing potential applications is important to showcase the usefulness of this approach and encourage readers to use it to study food-web structure or apply it to other types of biological networks. Good and bad predictions are both useful, either for assessing the predictive capacity of MaxEnt or understanding the ecological mechanisms driving food-web structure.

The authors mention that all code and data for reproduction purposes are available at the Open Science Framework and were made in open-source coding language Julia. So, beyond the opportunities for future applications of this work, they have made it accessible for replication via open-source software.

This is right. All code and data to reproduce this article are available online (osf.io/KT4GS).

We hope that our revised manuscript is now better suited for publication in PLoS Computational Biology. Reiterating our gratitude for the valuable feedback from both you and the reviewers, we are at your disposal to address any additional inquiries or information you may require.

Sincerely,

Francis Banville, Dominique Gravel, and Timothée Poisot
The Authors
Université de Montréal
1375 Thérèse-Lavoie-Roux Ave., Montreal, QC H2V 0B3, Canada
francis.banville@umontreal.ca
